# Outbreak of *Ralstonia pickettii* bacteraemia related to sterile distilled water in patients with haematological and oncological malignancies

**DOI:** 10.1017/S0950268826101526

**Published:** 2026-05-14

**Authors:** Sema Alp, Seyda Silan Okalin, Oya Ozlem Eren-Kutsoylu, Ayse Nur Sari-Kaygisiz, Gul Aygun, Cem Ergon, Aliye Cinar, Ulker Uysal, Berken Gur, Mehmet Ali Oktem

**Affiliations:** 1 Department of Infectious Diseases and Clinical Microbiology, Dokuz Eylul University Faculty of Medicine, Izmir, Türkiye; 2 BioIzmir - Izmir Health Technologies Development and Accelerator Research and Application Center, Dokuz Eylul University, Izmir, Türkiye; 3 Department of Medical Microbiology, Faculty of Medicine, Zubeyde Hanim Traning and Research Hospital, Baskent University, Izmir, Türkiye; 4 Infection Control Committee, Dokuz Eylul University Hospital, Izmir, Türkiye; 5 Department of Medical Microbiology, Faculty of Medicine, Dokuz Eylul University, Izmir, Türkiye; 6 Department of Medical Microbiology, Turkish Republic Ministry of Health, Hakkari State Hospital, Hakkari, Türkiye

**Keywords:** healthcare-associated infection, outbreak, *Ralstonia pickettii*, sterile distilled water

## Abstract

This study aimed to investigate a four-month *Ralstonia pickettii* outbreak by characterizing clinical and environmental isolates while also demonstrating the infection control measures taken to identify and eliminate the sources. *R. pickettii* was isolated from patients’ blood cultures, and an epidemiological investigation was initiated to determine the source of the outbreak. Clonal relatedness among clinical and environmental samples was assessed using pulsed-field gel electrophoresis. In total, *R. pickettii* was isolated from 14 patient blood cultures and 3 bottles of sterile distilled water. Between April 14 and May 15, six patients had positive port cultures, including four with positive peripheral cultures. The contaminated water was identified as the outbreak source, and its use was discontinued on May 15. Nevertheless, two additional cases occurred in June. Port catheter colonization with *R. pickettii* was considered to be linked to contaminated water used during chemotherapy preparation. A recall of port catheter patients who received chemotherapy in April–May identified six more cases. Pulsed-field gel electrophoresis revealed two pulsotypes among water isolates, and most clinical isolates were clonally related to clone 1. This investigation highlighted the role of contaminated sterile distilled water and colonized medical devices as potential sources of *R. pickettii* outbreaks.

## Introduction

*Ralstonia pickettii* was first described as *Pseudomonas pickettii* in 1973, and then, this bacterium was named *Burkholderia pickettii.* Afterwards, it was discovered that a distinct genus was involved in bacteria as *Ralstonia* in 1995 [1, 2]. *Ralstonia* is an aerobic, Gram-negative, and non-fermentative bacterium found in water and soil [2, 3].

*R. pickettii* is the most crucial species within the *Ralstonia* genus as an opportunistic pathogen and causes healthcare-associated infections [3]. *R. pickettii* has been isolated from clinical specimens, including blood, urine, cerebrospinal fluid, and tracheal aspirate [4, 5]. It may cause several infections, such as sepsis, pneumonia, meningitis, endocarditis, osteomyelitis, and endophthalmitis. These infections generally occur in immunosuppressive patient groups affected by heart disease, diabetes mellitus, chronic kidney disease, hydrocephalus, cystic fibrosis, and respiratory diseases[4, [6]–12]. *R. pickettii* can survive in drinking water supplies, hospital water supplies, or ultrapure water systems, which might cause contamination [3, 4, 13, 14]. Additionally, *R. pickettii* contaminates saline solution, sterile water for injections, intravenous products, and distilled water used for treatments, and causes healthcare-associated outbreaks [4, 7, 15, 16].

This study revealed a four-month-long *R. pickettii* outbreak in April 2020 and continued until July 2020 in a tertiary hospital in Izmir, Türkiye. During that time, *R. pickettii* isolates were isolated in the blood cultures from 14 critically ill patients hospitalized in the following wards: paediatric haematology clinic, haematological stem cell transplant unit, haematology department, and oncology outpatient chemotherapy unit. Also, in this study, we have indicated infection control measures following monitoring studies to determine the outbreak origin that allowed to end it.

## Materials and methods

### Study setting

Dokuz Eylul University Hospital is a tertiary care institution with a capacity of 1100 beds, located in Izmir, Türkiye. Within the hospital complex, the haematology and oncology hospital encompasses a 54-bed facility that includes a haematopoietic stem cell transplantation unit, as well as dedicated haematology and oncology clinics. Additionally, paediatric haematology services are provided at the 21-bed paediatric haematology clinic within the Dokuz Eylul University Children’s Hospital. The outpatient chemotherapy unit plays a significant role in patient care, managing approximately 20000 admissions annually for chemotherapy administration. In the hospital’s chemotherapy drug preparation unit, cytotoxic agents are reconstituted using commercial sterile distilled water via an automated system under pharmacy supervision, ensuring both safety and standardization in drug preparation processes.

Infection prevention efforts at Dokuz Eylul University Hospital are coordinated by the Infection Control Committee, which has been actively operating since its establishment in 1992. At our hospital, the Infection Control Committee consists of three nurses, two specialists in clinical microbiology and infectious diseases, and two medical microbiology specialists in the molecular epidemiology laboratory, which plays a central role in supporting the hospital’s infection control strategies. This laboratory is conducting molecular analyses during outbreak investigations, enabling the identification of clonal relationships among pathogens through techniques such as pulsed-field gel electrophoresis (PFGE). The laboratory analyzes the genetic similarity between isolates to identify the source and spread of infections, helping to implement effective control measures. Furthermore, collaboration between the Infection Control Committee and the molecular epidemiology laboratory supports evidence-based surveillance. This cooperation also enhances patient safety and contributes to the monitoring of antimicrobial resistance.

This outbreak report was prepared in accordance with the ORION (Outbreak Reports and Intervention Studies of Nosocomial Infection) statement guidelines for reporting nosocomial infection outbreaks and intervention studies [17].

To standardize the outbreak investigation, the following case definitions were applied: Confirmed bloodstream infection (BSI) was defined as the presence of *R. pickettii* in peripheral blood cultures (either alone or together with positive port catheter cultures), accompanied by clinical symptoms.

Transient bacteraemia was defined as the presence of clinical symptoms – specifically chills and rigors – occurring during chemotherapy administration, with subsequent isolation of *R. pickettii* from port catheter blood cultures.

Port catheter colonization was defined as clinical stability and absence of symptoms in a patient whose *R. pickettii* growth was restricted solely to port catheter blood cultures, while peripheral blood cultures remained negative.

Environmental source was defined as any sample of commercial sterile distilled water, normal saline solution, or reconstituted drug solution that yielded *R. pickettii* during the investigation period, specifically those identified through traceability of shared batch or lot numbers.

### Clinical isolates, environmental sampling, and microbiological investigation

Peripheral and port catheter blood cultures were obtained from all patients. Catheter tip cultures were performed in only two patients. The absence of systematic catheter tip sampling in the remaining cases was mainly attributable to the retrospective design of the study and the substantial clinical workload and organizational disruptions associated with the COVID-19 pandemic. All isolates were identified by matrix-assisted laser desorption/ionization – time of flight mass spectrometry (MALDI-TOF MS) (Bruker Biotyper; Bruker Daltonics, Bremen, Germany) after 48 h of incubation on blood agar and were stored in brain-heart infusion broth with 10% glycerol at −80^°^C for further analysis.

For the purpose of outbreak investigation and follow-up studies, the infection control committee visited the relevant clinical units and the chemotherapy preparation area within the hospital pharmacy. Comprehensive traceability investigation was performed by reviewing pharmacy and nursing records to identify the drug names, brands, and batch/lot numbers of all administered medications and diluents were identified. The analysis revealed that the universal commonality across all affected patients was the use of specific batches of commercial sterile distilled water and normal saline for drug reconstitution and administration. Subsequently, samples of these medications, commercial sterile distilled water, and saline solutions were collected, cultured, and subjected to microbiological analysis to identify the potential source of contamination. Bacterial growth on blood agar was observed after 24 and 48 h of incubation, and the isolates were identified using MALDI-TOF MS. All confirmed isolates were preserved under the previously described conditions for further analysis.

### Epidemiological relatedness of *R. pickettii* isolates

PFGE analysis was performed in two stages using the CHEF-DR III System (Bio-Rad, USA). In the first stage, PFGE was carried out on four early clinical isolates (two actively reported cases and two retrospectively identified isolates) to determine their clonal relatedness. This initial evaluation confirmed that the isolates were genetically identical, and the earliest isolate was subsequently selected as the representative strain for further comparisons. In the second stage, PFGE was performed on the representative strain, additional clinical port blood culture isolates, and environmental samples to assess the overall clonal relationship between clinical and environmental sources. In PFGE studies, DNA was digested with *Xba*I according to the ‘CDC Standard Operating Procedure for PulseNet PFGE of Cronobacter Species’ [18] with the following modifications for electrophoresis conditions on CHEF DR III: initial switch time is 1.8 s, final switch time is 25 s, voltage is 6 V, included angle is 120°, and run time is 20 h.

The dendrogram was constructed based on PFGE banding patterns using the UPGMA (Unweighted Pair Group Method with Arithmetic Mean) clustering algorithm implemented in the PyElph 1.4 software tool [19] and was subsequently visualized using the Interactive Tree of Life (iTOL) platform [20].

### Antimicrobial susceptibility test

The antimicrobial susceptibility of representative isolates was assessed using the disk diffusion method. Bacterial suspensions were prepared from fresh colonies of *R. pickettii* isolates grown on blood agar plates after overnight incubation. The turbidity was adjusted to a 0.5 McFarland standard using a nephelometer. Suspensions were evenly spread onto Mueller-Hinton agar plates (RTA Laboratories, Türkiye) using sterile swabs. Antibiotic disks, including meropenem, imipenem, ciprofloxacin, levofloxacin, ceftazidime, piperacillin-tazobactam, tobramycin, and amikacin, were carefully placed on the inoculated plates using sterile forceps. Plates were incubated at 37°C for 18 h under standard atmospheric conditions. The results of the disk diffusion test were evaluated in accordance with the breakpoints established by the European Committee on Antimicrobial Susceptibility Testing (EUCAST), using *Pseudomonas* spp. as the reference organism.

### Ethics

The study was approved by the Dokuz Eylul University Non-Interventional Research Ethics Committee (date and no: 6 January 2022 – 2022/20-21).

## Results

### Clinical isolates, environmental sampling, and microbiological investigation

The first reported *R. pickettii* growth was isolated from the blood sample of an 11-year-old female patient hospitalized in the paediatric haematology clinic (index case, case 3). After the case was reported to the Infection Control Committee, the same species was subsequently isolated from 13 additional patients hospitalized in the hematopoietic stem cell transplant unit, the haematology clinic, and the outpatient chemotherapy unit. The period of isolation for the 14 isolates was from April 2020 to July 2020. The Infection Control Committee recorded patients’ demographic data, bacterial isolation dates, and antimicrobial therapy (Table 1). Four patients with hematologic malignancy and positive cultures from both peripheral blood and port catheters (cases 1, 3, 4, and 6) presented with clinical symptoms of infection, primarily neutropenic fever, confirming the presence of *R. pickettii*-related true bloodstream infections. These patients had significantly lower absolute neutrophil counts (ANCs < 500 / μL) and required intensive management with broad-spectrum antibiotics, specifically meropenem. In contrast, the remaining 10 patients, whose positive cultures were restricted solely to the port catheter, generally remained clinically stable. Among these 10 patients, cases 2, 5, 7, 8, 9, 13, and 14 were defined as having transient bacteraemia, as they exhibited clinical symptoms – specifically chills and rigours – only during chemotherapy administration. The remaining three patients were entirely asymptomatic and were identified as having port catheter colonization rather than active infection. The majority of patients in these two groups received empirical antibiotic therapy with ciprofloxacin. Catheter-tip culture was performed in only two patients (cases 7 and 13), from which *R. pickettii* was subsequently isolated. During the *R. pickettii* outbreak, the COVID-19 pandemic was concurrently ongoing. All patients were screened for SARS-CoV-2, and two of them tested positive (cases 2 and 3). Case 2, who had a significant medical history of bronchial asthma, developed COVID-19–related pneumonia and was treated with levofloxacin. Additionally, a urinary tract infection caused by *Klebsiella pneumoniae* was documented in one patient (case 13).

**Table 1.** Demographic and clinical data of patients affected during the *R. pickettii* outbreak Table 1. long description.

Patient	Age	Gender	Unit	Malignity	Chemotherapy (14 April–15 May)	Isolation date	White Blood Cell / μL (Absolute neutrophil %)	Duration of fever	Clinical status	Antimicrobial therapy	Date of port catheter placement and removal
1	54	F	Haematology	AML	Idarubicin Cytosine arabinoside	14 April	400 (3%)	Until the catheter was removed	Febrile neutropenia BSI	Meropenem	8 April 2020 and 24 April 2020
2	65	F	Outpatient chemotherapy	Breast	Docetaxel Trastuzumab Pertuzumab	21 April	20100 (97%)	During chemotherapy	Colonized transient bacteraemia	Levofloxacin	24 October 2019 and 7 May 2020
3	11	F	Paediatrics haematology	AML	Idarubicin Cytosine arabinoside	10 May	900 (46%)	Until the catheter was removed	Febrile neutropenia BSI	Meropenem	20 March 2020 and 27 May 2020
4	53	M	HSCTU	ALL (Refracter)	Idarubicin -FLAG	10 May	800 (90%)	Until the catheter was removed	Febrile neutropenia, BSI	Meropenem	17 March 2020 and 16 May 2020
5	40	F	Outpatient chemotherapy	Breast	Trastuzumab	15 May	5100 (55%)	During chemotherapy	Colonized Transient bacteraemia	Ciprofloxacin	6 May 2018 and 3 June 2020
6	3	F	Paediatrics haematology	ALL	Doxorubicin Vincristine	15 May	1100 (32%)	Until the catheter was removed	Febrile neutropenia BSI	Meropenem	17 October 2019 and 6 June 2020
7	40	F	Outpatient chemotherapy	Breast	Trastuzumab	18 June	4700 (60%)	During chemotherapy	Colonized transient bacteraemia	Ciprofloxacin	8 January 2020 and 8 June 2020
8	58	F	Outpatient chemotherapy	Breast	Trastuzumab	27 June	7400 (65%)	During chemotherapy	Colonized transient bacteraemia	Ciprofloxacin	1 September 2018 and 7 July 2020
9	48	F	Outpatient chemotherapy	Breast	Trastuzumab	2 July	5400 (48%)	On the same day following chemotherapy	Colonized transient bacteraemia	Ciprofloxacin	21 December 2019 and 7 July 2020
10	55	F	Outpatient chemotherapy	Breast	Trastuzumab	2 July	5500 (60%)	Absent	Colonized	Ciprofloxacin	17 December 2019 and 7 July 2020
11	55	F	Outpatient chemotherapy	Breast	Trastuzumab	3 July	6600 (58%)	Absent	Colonized	Ciprofloxacin	27 May 2019 and 7 July 2020
12	46	F	Outpatient Chemotherapy	Breast	Trastuzumab	6 July	7200 (56%)	Absent	Colonized	Ciprofloxacin	31 April 2019 and 4 August 2020
13	63	F	Outpatient chemotherapy	Breast	Trastuzumab	13 July	5000 (61%)	On the same day following chemotherapy	Colonized transient bacteraemia	Ciprofloxacin	16 August 2018 and 5 August 2020
14	65	F	Outpatient chemotherapy	Breast	Trastuzumab	16 July	10200 (75%)	On the same day following chemotherapy	Colonized transient bacteraemia	Ciprofloxacin	20 February 2020 and 4 August 2020

ALL, acute lymphoblastic leukaemia; AML, acute myeloid leukaemia; BSI, blood stream infection; F, female; FLAG, fludarabine, high-dose cytarabine; HSCTU, haematopoietic stem cell transplantation unit; M, male.

Commonly used drugs were identified through pharmacy records, and microbiological cultures were performed on antipyretic, antiemetic, and chemotherapeutic agents, as well as on sterile distilled water and saline samples. *R. pickettii* was detected in the three sterile distilled water bottles that had been used for the dilution of chemotherapy drugs namely idarubicin, trastuzumab, and doxorubicin (Table 1). *R. pickettii* was not detected in any of the other environmental specimens collected.

### Epidemiological relatedness of *R. pickettii* isolates

**Figure 1.**
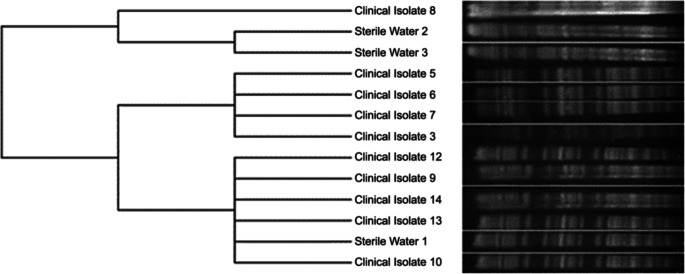
PFGE-based dendrogram showing the clonal relationship of *R. pickettii* isolates. Clinical isolate 3 is included as the representative isolate for the initial identical cluster (cases 1–4, previously confirmed to be 100% similar). All other labels (clinical isolates 5–14 and sterile water 1–3) represent unique, independent isolates. Fig. 1 long description.

PFGE was performed on a total of 14 clinical isolates and 3 environmental samples in two stages of analysis to confirm the source of the outbreak. Initial PFGE analysis of four *R. pickettii* clinical isolates collected between April and May 2020 revealed a single clonal lineage with an identical pulsotype. Notably, the first reported *R. pickettii* isolate, obtained from the blood culture of a paediatric patient in the paediatric haematology clinic (case 3), was the index isolate that triggered the outbreak notification and was selected as the representative strain for subsequent analysis. Environmental sampling was performed, yielded *R. pickettii* from three distinct bottles of sterile distilled water. A second-stage PFGE analysis was subsequently conducted, incorporating the representative isolate (clinical isolate 3), ten additional clinical isolates, and the three water-derived isolates. These results demonstrated that all but one clinical isolate (clinical isolate 8) shared the same pulsotype (clone 1) as the sample from sterile distilled water 1. Conversely, sterile distilled water samples 2 and 3 exhibited a distinct pulsotype (clone 2), which was identical to clinical isolate 8. One clinical isolate remained untypable due to experimental failure. The clonal relationships identified in the second PFGE stage are shown via a dendrogram (Figure 1).

### Antimicrobial susceptibility test

The antimicrobial resistance results obtained using the disk diffusion method and evaluated based on the breakpoints recommended for *Pseudomonas* species are presented in Table 2.

**Table 2.** The antimicrobial susceptibility pattern of *R. pickettii* Table 2. long description.

Antibiotics used for disk-diffusion test	*R. pickettii* (clone 1)		*R. pickettii* (clone 2)	
	Breakpoints (mm)	Susceptibility	Breakpoints (mm)	Susceptibility
Meropenem/MEM10	28	S	30	S
Imipenem/IMP10	30	I	30	I
Ciprofloxacin/CIP5	28	I	28	I
Levofloxacin/LEV5	28	I	32	I
Ceftazidime/CAZ10	15	R	18	I
Piperacillin-Tazobactam/TZP36	28	I	32	I
Tobramycin/TOB10	15	R	6	R
Amikacin/AK30	18	S	16	S

### Follow-up and infection control studies

**Figure 2.**
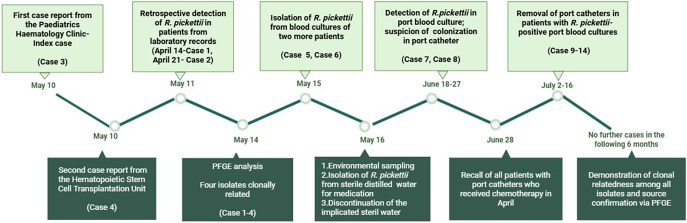
Timeline of the *R. pickettii* outbreak investigation and control measures (timeline created with BioRender). Fig. 2 long description.

The outbreak investigation was formally initiated on May 10 following the clinical microbiology laboratory’s notification of *R. pickettii* in the blood cultures of two active patients (cases 3 and 4). A subsequent retrospective audit of laboratory records on May 11 revealed two additional, previously unreported isolations from April 14 and April 21 (cases 1 and 2). To investigate a potential clonal relationship, the first four available isolates were subjected to PFGE analysis on May 14, which confirmed 100% genetic similarity among them, thereby verifying a clonal outbreak. Following the confirmation of clonal similarity, environmental samples were collected, and *R. pickettii* was successfully isolated from three different bottles of commercial sterile distilled water used for medication preparation. Although the use of commercial sterile distilled water was discontinued immediately after its identification as the source, *R. pickettii* continued to be isolated from the blood cultures of two additional patients (cases 7 and 8) in June 2020. In cases 7 and 8, clinical manifestations such as chills and rigours during chemotherapy administration necessitated the collection of blood cultures from both the port catheter and peripheral sites. Subsequent microbiological analysis led to the isolation of *R. pickettii* from the port catheter blood cultures of these patients. A retrospective evaluation of these cases revealed that the two affected patients, both diagnosed with breast cancer, had received chemotherapy via port catheters during April and May. This finding suggested that the patients had been treated with medications diluted with contaminated sterile distilled water, leading to the colonization of the port catheters by R.pickettii. All patients with breast cancer who had a port catheter and received chemotherapy in April and May were recalled to the hospital. Recalled patients underwent clinical evaluation, and both peripheral and port catheter blood cultures were obtained for laboratory analysis. While cases 9, 13, and 14 presented with transient bacteraemia, cases 10, 11, and 12 remained entirely asymptomatic. Notably, *R. pickettii* was isolated from the port catheter blood cultures of all six patients. Following these findings, the port catheters were removed in the interventional radiology unit. Empirical ciprofloxacin therapy was administered to these patients until the successful removal of the catheters. Catheter tip cultures were performed in only two patients, and both yielded *R. pickettii.* Consequently, the assessment of catheter involvement in the remaining cases was primarily based on temporal association and the results of paired blood cultures. A timeline of the outbreak and investigative process is presented in Figure 2.

## Discussion

*R. pickettii* is considered the most clinically significant species within the *Ralstonia* genus due to its role as an opportunistic pathogen, frequently associated with healthcare-related infections and outbreaks. It has been reported to contaminate saline solutions, sterile water, and intravenous products, thereby contributing to healthcare-associated outbreaks [3, 4, 7, 15, 16]. Generally, the specialized 0.2-μm size filters are used in the filtration processes of these types of water systems and solutions. Since some *R. pickettii* isolates may be small enough to pass through 0.2-μm size filters, they can contaminate sterile solutions [21]. However, this species also causes pseudo-outbreaks as a consequence of the use of media and agar, which are prepared using water or solutions contaminated with *R. pickettii* [22] Furthermore, *R. picketti* has also been shown to survive in disinfectants such as chlorhexidine [23].

In the present study, an outbreak caused by *R. pickettii* was investigated, and its source was identified. Several previous studies have also reported *R. pickettii*-related outbreaks [7, 16, 24, 25]. However, many studies that reported *R. pickettii*–related outbreaks have been unable to identify their source [24, 26, 27]. Although the sterile distilled water was identified as the primary source of the outbreak in our study, and its use was discontinued, the outbreak persisted for an extended period of four months. The persistence of the outbreak was due to the colonization of *R. pickettii* within the port catheters. Biofilm, a significant virulence factor for *R. pickettii*, facilitates bacterial colonization of catheters, and as reported in studies, the organism can colonize catheters [28–30]. Therefore, our results indicate that contamination of catheters or other invasive medical materials should always be considered in any *R. pickettii* infections or outbreaks.

In our study, the clonal relationship between clinical and environmental isolates was investigated using PFGE. The PFGE protocol was optimized for *R. pickettii*, and the commercial sterile distilled water was identified as the primary source of the outbreak. In line with our study, a few studies have used PFGE to investigate healthcare-associated outbreaks caused by *R. pickettii* [6, [7], 27]. Our PFGE results also showed two distinct pulsotypes from three different sterile distilled water samples. In the study by Moreira et al., it was demonstrated that contamination by *R. pickettii* can be associated with more than one pulsotype [7].

In recent years, several *R. pickettii* outbreak investigations have successfully identified the source of contamination, often involving sterile water, saline solutions, or other medical products [31–33]. These findings suggest an increasing awareness and improvements in outbreak investigation strategies. However, one of the major challenges in *R. pickettii*–related outbreaks is the considerable time required to identify the source. This delay often prevents the timely implementation of effective control measures and prolongs the outbreak. Although *R. pickettii* is generally susceptible to antimicrobials, it can cause healthcare-associated infections and outbreaks. Such infections are more commonly associated with multidrug-resistant organisms, including *Acinetobacter baumannii*, *K. pneumoniae*, *Pseudomonas aeruginosa*, *Proteus mirabilis*, and *Escherichia coli.* These pathogens are capable of transmitting resistance genes to other bacteria via transposons or horizontal gene transfer. The ability of *R. pickettii* to persist for prolonged periods in hospitalized patients and in the hospital environment, may increase the likelihood of acquiring such resistance determinants [34].

Although *R. pickettii* is considered to have relatively low intrinsic virulence, its ability to form biofilms significantly enhances its pathogenic potential. The bacterium can colonize invasive medical devices, leading to persistent infections. This is particularly concerning in immunocompromised patients, who are more likely to require catheterization. Such colonization can pose a significant risk for systemic infections, especially in patients with prolonged hospital stays or underlying comorbidities. Once colonized by opportunistic, biofilm-forming bacteria such as *R. pickettii*, catheter removal becomes necessary to control the infection. However, the insertion and removal of port catheters are technically demanding procedures. Port catheters are usually removed only when they are no longer clinically necessary or when complications such as infection occur. In the present study, some patients had received port catheters nearly two years before the outbreak.

The present study has several notable strengths. First, the source of the outbreak was successfully identified. Second, the clonal relationship among the isolates was conclusively demonstrated through PFGE, providing strong molecular evidence. In addition, patients suspected of port catheter colonization were promptly re-evaluated for catheter removal, thereby preventing potential systemic complications.

Our study has several limitations. Microbiological confirmation through catheter-tip cultures was not systematically performed for all patients; however, *R. pickettii* was isolated from the catheter tip of two patients, providing microbiological support for the observed findings. Furthermore, the resolution of the outbreak following the removal of port catheters suggests a temporal association. The absence of new cases during the subsequent 6-month follow-up period is consistent with the hypothesis that these catheters acted as a reservoir for *R. pickettii* during the contamination event.

## Conclusions

Outbreaks caused by *R. pickettii* are rare in healthcare settings; however, in the event of an outbreak, contamination of the hospital environment, medical equipment, or sterile solutions should be suspected. Timely epidemiological investigations are essential for the detection of sources and control of outbreaks.

## Data Availability

The study is based on anonymized patient data and microbiological analyses. All data are included within this article. Any queries regarding the outbreak analysis may be directed to the authors.

## Table 1 Long description

The table contains 14 rows, each for a patient, and 12 columns: Patient, Age, Gender, Unit, Malignity, Chemotherapy (14 April to 15 May), Isolation date, White blood cells counts per microliter (Absolute neutrophil percent), Duration of fever, Clinical status, Antimicrobial therapy, and Date of port catheter placement and removal. Patient 1: 54, female, Haematology, AML, Idarubicin and Cytosine arabinoside, isolated 14 April, neutrophils 400 (3 percent), fever until catheter removal, febrile neutropenia B S I, treated with Meropenem, catheter placed 8 April 2020 and removed 24 April 2020. Patient 2: 65, female, Outpatient chemotherapy, Breast, Docetaxel, Trastuzumab, Pertuzumab, isolated 21 April, White blood cells counts per microliter 20100 (neutrophils 97 percent), fever during chemotherapy, colonized transient bacteraemia, Levofloxacin, catheter placed 24 October 2019 and removed 7 May 2020. Patient 3: 11, female, Paediatrics haematology, AML, Idarubicin and Cytosine arabinoside, isolated 10 May, White blood cells counts per microliter 900 (neutrophils 46 percent), fever until catheter removal, febrile neutropenia B S I, Meropenem, catheter placed 20 March 2020 and removed 27 May 2020. Patient 4: 53, male, H S C T U, ALL (refractory), Idarubicin-F L A G, isolated 10 May, white blood cells counts per microliter 800 (neutrophils 90 percent), fever until catheter removal, febrile neutropenia B S I, Meropenem, catheter placed 17 March 2020 and removed 16 May 2020. Patient 5: 40, female, Outpatient chemotherapy, Breast, Trastuzumab, isolated 15 May, white blood cells counts per microliter 5100 (neutrophils 55 percent), fever during chemotherapy, colonized transient bacteraemia, Ciprofloxacin, catheter placed 6 May 2018 and removed 3 June 2020. Patient 6: 3, female, Paediatrics haematology, ALL, Doxorubicin and Vincristine, isolated 15 May, white blood cells counts per microliter 1100 (neutrophils 32 percent), fever until catheter removal, febrile neutropenia B S I, Meropenem, catheter placed 17 October 2019 and removed 6 June 2020. Patient 7: 40, female, Outpatient chemotherapy, Breast, Trastuzumab, isolated 18 June, white blood cells counts per microliter 4700 (neutrophils 60 percent), fever during chemotherapy, colonized transient bacteraemia, Ciprofloxacin, catheter placed 8 January 2020 and removed 8 June 2020. Patient 8: 58, female, Outpatient chemotherapy, Breast, Trastuzumab, isolated 27 June, white blood cells counts per microliter 7400 (neutrophils 65 percent), fever during chemotherapy, colonized transient bacteraemia, Ciprofloxacin, catheter placed 1 September 2018 and removed 7 July 2020. Patient 9: 48, female, Outpatient chemotherapy, Breast, Trastuzumab, isolated 2 July, white blood cells counts per microliter 5400 (neutrophils 48 percent), fever on same day after chemotherapy, colonized transient bacteraemia, Ciprofloxacin, catheter placed 21 December 2019 and removed 7 July 2020. Patient 10: 55, female, Outpatient chemotherapy, Breast, Trastuzumab, isolated 2 July, white blood cells counts per microliter 5500 (neutrophils 60 percent), fever absent, colonized, Ciprofloxacin, catheter placed 17 December 2019 and removed 7 July 2020. Patient 11: 55, female, Outpatient chemotherapy, Breast, Trastuzumab, isolated 3 July, white blood cells counts per microliter 6600 (neutrophils 58 percent), fever absent, colonized, Ciprofloxacin, catheter placed 27 May 2019 and removed 7 July 2020. Patient 12: 46, female, Outpatient chemotherapy, Breast, Trastuzumab, isolated 6 July, white blood cells counts per microliter 7200 (neutrophils 56 percent), fever absent, colonized, Ciprofloxacin, catheter placed 31 April 2019 and removed 4 August 2020. Patient 13: 63, female, Outpatient chemotherapy, Breast, Trastuzumab, isolated 13 July, white blood cells counts per microliter 5000 (neutrophils 61 percent), fever on same day after chemotherapy, colonized transient bacteraemia, Ciprofloxacin, catheter placed 16 August 2018 and removed 5 August 2020. Patient 14: 65, female, Outpatient chemotherapy, Breast, Trastuzumab, isolated 16 July, white blood cells counts per microliter 10200 (neutrophils 75 percent), fever on same day after chemotherapy, colonized transient bacteraemia, Ciprofloxacin, catheter placed 20 February 2020 and removed 4 August 2020. Table footnotes define abbreviations: ALL is acute lymphoblastic leukaemia, AML is acute myeloid leukaemia, B S I is blood stream infection, F is female, F L A G is fludarabine high-dose cytarabine, H S C T U is haematopoietic stem cell transplantation unit, M is male.

Navigate back to Table 1.

## Table 2 Long description

The table has five columns: antibiotics, breakpoints and susceptibility for R. pickettii clone 1, and breakpoints and susceptibility for clone 2. From top to bottom: Meropenem/MEM10 shows 28 mm S for clone 1 and 30 mm S for clone 2. Imipenem/IMP10 is 30 mm I for both clones. Ciprofloxacin/CIP5 is 28 mm I for both. Levofloxacin/LEV5 is 28 mm I for clone 1 and 32 mm I for clone 2. Ceftazidime/CAZ10 is 15 mm R for clone 1 and 18 mm I for clone 2. Piperacillin-Tazobactam/TZP36 is 28 mm I for clone 1 and 32 mm I for clone 2. Tobramycin/TOB10 is 15 mm R for clone 1 and 6 mm R for clone 2. Amikacin/AK30 is 18 mm S for clone 1 and 16 mm S for clone 2. S indicates susceptible, I intermediate, R resistant.

Navigate back to Table 2.

## Figure 1 Long description

The dendrogram starts at the left with a single root, splitting into two main branches. The upper branch contains Clinical Isolate 8, Sterile Water 2, and Sterile Water 3, each with similar banding patterns. The lower branch further divides into Clinical Isolate 5, Clinical Isolate 6, Clinical Isolate 7, and Clinical Isolate 3, which cluster closely together, indicating high similarity. Below this cluster, Clinical Isolate 12, Clinical Isolate 9, Clinical Isolate 14, Clinical Isolate 13, Sterile Water 1, and Clinical Isolate 10 are arranged sequentially, each with unique banding profiles. The banding patterns to the right of each label represent the P F G E profiles, with visible differences in band intensity and position, highlighting clonal relationships and genetic diversity among the isolates.

Navigate back to Figure 1.

## Figure 2 Long description

From left to right, the timeline begins at May 10 with the first case report from the Paediatrics Haematology Clinic (Case 3) in an upper box, and below it, the second case report from the Hematopoietic Stem Cell Transplantation Unit (Case 4). On May 11, retrospective detection of R. pickettii in patients from laboratory records is noted (April 14–Case 1, April 21–Case 2) in the upper box, with PFGE analysis showing four isolates clonally related (Cases 1–4) in the lower box. On May 14, isolation of R. pickettii from blood cultures of two more patients (Cases 5, 6) is shown above, with environmental sampling, isolation of R. pickettii from sterile distilled water, water disinfection, and discontinuation of the implicated sterile water listed below. On May 16, detection of R. pickettii in port blood culture and suspicion of colonization in port catheter (Cases 7, 8) is shown above, with recall of all patients with port catheters who received chemotherapy in April below (June 18–27). On June 28, removal of port catheters in patients with R. pickettii-positive port blood cultures (Cases 9–14) is shown above, and below, demonstration of clonal relatedness among all isolates and source confirmation via PFGE. The timeline ends at July 2–16 with no further cases in the following 6 months.

Navigate back to Figure 2.
